# Machine learning surrogates for surface complexation model of uranium sorption to oxides

**DOI:** 10.1038/s41598-024-57026-w

**Published:** 2024-03-19

**Authors:** Chunhui Li, Elijah O. Adeniyi, Piotr Zarzycki

**Affiliations:** 1 Energy Geosciences Division, Lawrence Berkeley National Laboratory, Berkeley, CA USA; 2 Department of Earth Sciences, Montana State University, Bozeman, MT USA

**Keywords:** Pollution remediation, Natural hazards

## Abstract

The safety assessments of the geological storage of spent nuclear fuel require understanding the underground radionuclide mobility in case of a leakage from multi-barrier canisters. Uranium, the most common radionuclide in non-reprocessed spent nuclear fuels, is immobile in reduced form (U(IV) and highly mobile in an oxidized state (U(VI)). The latter form is considered one of the most dangerous environmental threats in the safety assessments of spent nuclear fuel repositories. The sorption of uranium to mineral surfaces surrounding the repository limits their mobility. We quantify uranium sorption using surface complexation models (SCMs). Unfortunately, numerical SCM solvers often encounter convergence problems due to the complex nature of convoluted equations and correlations between model parameters. This study explored two machine learning surrogates for the 2-pK Triple Layer Model of uranium retention by oxide surfaces if released as U(IV) in the oxidizing conditions: random forest regressor and deep neural networks. Our surrogate models, particularly DNN, accurately reproduce SCM model predictions at a fraction of the computational cost without any convergence issues. The safety assessment of spent fuel repositories, specifically the migration of leaked radioactive waste, will benefit from having ultrafast AI/ML surrogates for the computationally expensive sorption models that can be easily incorporated into larger-scale contaminant migration models. One such model is presented here.

## Introduction

Development of sustainable, low-carbon energy production technologies is of paramount importance due to growing energy demand in the realm of the global warming^1,2^. Nuclear power emerges as a promising solution, offering stable and clean energy while minimizing $\hbox {CO}_2$ emissions to mitigate climate change^3,4^. However, the safe treatment and disposal of nuclear waste remain a challenge^5,6^.

**Figure 1:**
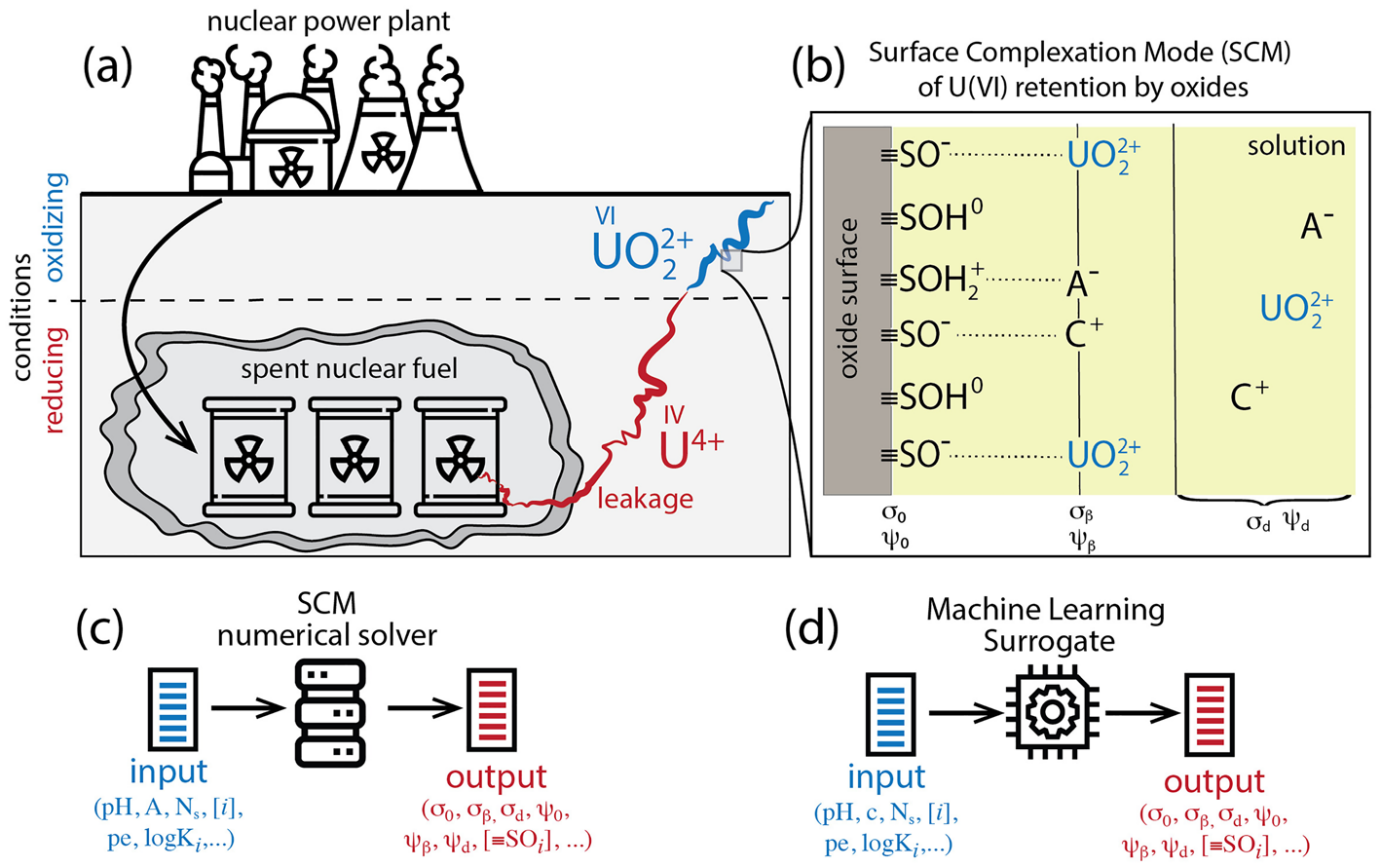
Schematic illustration of the hypothetical leakage of spent nuclear fuel from geological storage, mobilization of uranium (U) in oxidizing subsurface conditions, and retention by sorption to oxides minerals (**a**). In panel (**b**), we show a schematic diagram of the 2-pK Triple Layer Model (TLM) of the oxide/U-containing solution interface: 0-plane at the mineral surface, d-plane at diffuse layer and $\beta$-plane is the layer where electrolyte ions adsorb non-specifically. $\hbox {C}^+$ and $\hbox {A}^-$ represent the electrolyte cations and anions (i.e., $\hbox {Na}^+$, $\hbox {Cl}^-$). In panels (**c**, **d**), we show the numerical approach to predict thermodynamic properties of the mineral/electrolyte interface and the extent of ion sorption (**c**) and its Machine Learning surrogate - described in this report (**d**). The input contains a description of system conditions such as pH, ions concentrations ([*i*], *i*= $\hbox {C}^+$, U), surface area (A), site density ($\hbox {N}_s$), and ion/proton affinity constants (log $\hbox {K}_i$). The output contains charge densities and electrostatic potentials at the surface, $\beta$ and diffuse layers ($\sigma _0, \sigma _\beta , \sigma _d, \psi _0, \psi _\beta , \psi _d$) and concentration of various types of surface species ([$\equiv \hbox {SO}_i$]). Despite oxidizing conditions, we also consider a special case in which U(IV) is adsorbed strongly to the surface, and thus, it is protected against oxidation in the solution.

Geological repositories are considered to store spent nuclear fuel^7,8^. This approach involves multi-barrier isolation of the radioactive waste, with the host rock formation being the last barrier^9,10^. The safety assessment of such repositories requires knowledge of the mobility of radionuclides that could be released to the surrounding environment (e.g., nuclear waste leakage, Fig. 1a) and upward transport to groundwater and soils remain a concern^11–13^.

To assess the safety of the spent nuclear waste disposal sites in case of leakage, one must quantify the strength and extent of radionuclides retention by mineral/soil matrix. This study focused on uranium because it is the most abundant radionuclide in non-reprocessed spent nuclear fuel and is highly mobile if present in the oxidized form. Most of the repositories are planned deep underground, where reducing conditions prevail. In these conditions, uranium is present in a reduced U(IV) form and is strongly attracted to negatively charged organic and inorganic surfaces; thus, it is immobile. In the event of a leakage, released U(IV) could be retained within the leakage zone via sorption to mineral surfaces^14,15^. Particles with adsorbed U(IV) could be transported upward to oxic conditions^16–18^. Oxidized uranium, U(VI), remains stable in solution and is considered an environmental threat if released into groundwater^19^.

Our work refers to a hypothetical leakage of spent nuclear fuel from multibarrier containers, followed by its upward transport to the subsurface, where oxidation reactions mobilize uranium transport by transforming immobile uranium (IV) into mobile uranium (VI) (Fig. 1a). We consider a redox equilibrium between reduced and oxidized uranium but assume a positive redox potential (pe = 0.5). Consequently, uranium (VI) is a dominant oxidation state, and U is present as $\hbox {UO}_2^{2+}$ ion. However, if U(IV) is strongly adsorbed to the mineral surfaces, it may remain as U(IV), protected from oxidation in the solution to U(VI). In this study, we consider a broad range of U(IV) and U(VI) sorption affinities to account for the fact that uranium may remain as U(IV) adsorbed to the mineral surfaces despite oxidative conditions.

Uranium mobility and environmental toxicity in the event of a compromised nuclear waste repository depend on its interactions with the surrounding soil/rock and their constituents. The key environmental descriptors controlling uranium sorption are pH, redox conditions, salinity, and presence of complexing ligands/mineral surfaces^14,15,20^. The retention by mineral surfaces can be calculated using the interfacial speciation models known as surface complexation models (SCMs)^20–23^. One of the challenges of applying SCM models to estimate uranium retention by mineral surfaces is the complexity of convoluted and coupled equations that need to be numerically solved^20,24^. This translates to convergence problems and computational costs^25^, requiring inputs from domain expertise to resolve the issue.

Recently, we demonstrated the effectiveness of applying traditional machine learning methods while developing surface composite models (SCMs)^26^. In particular, we employed an ensemble of random forest regressors to accurately predict SCM parameters, simplifying and enhancing the SCM development process. In this study, we curated extensive datasets, comprising more than one million data points, covering diverse conditions of uranium sorption on oxides using advanced numerical SCM solvers. Recognizing the advantages of deep learning for large datasets, in addition to traditional machine learning methods, we explored a deep neural network model. We aim to create a cost-effective yet accurate surrogate model with minimal human input for predicting uranium sorption to oxides across a broad range of surface conditions. This effort contributes to the safety assessment of spent fuel repositories by introducing a class of artificial intelligence/machine learning (AI/ML) surrogates capable of predicting uranium retention for a broader range of subsurface conditions at a fraction of the computational cost compared to analytical models.

## Results

We generated a comprehensive dataset comprising 1,589,411 entries via a traditional SCM solver^27^. Each entry consists of 12 system descriptors: pH, ions concentrations ([*i*], where *i* = $\hbox {C}^+$,U), surface area (A), site density ($\hbox {N}_s$), and ion/proton affinity constants ($\hbox {logK}_i$), which will be used as inputs to the surrogate model (see Table S3, Supporting Information). The information of charge densities and electrostatic potentials at the surface, $\beta$ and diffuse layers, and concentration of various surface species ([$\equiv$ $\hbox {SO}_i$] were then extracted and used as target values when developing uranium sorption surrogate models. Subsequently, We developed two uranium sorption surrogate models: a multioutput regressor composed of an ensemble of random forests (RF-surrogate) and a deep neural network model constructed from six fully connected layers (DNN-surrogate). Both surrogate models have the same input parameter format with 12 system descriptors as the numerical SCM solver in generating the dataset.

**Figure 2:**
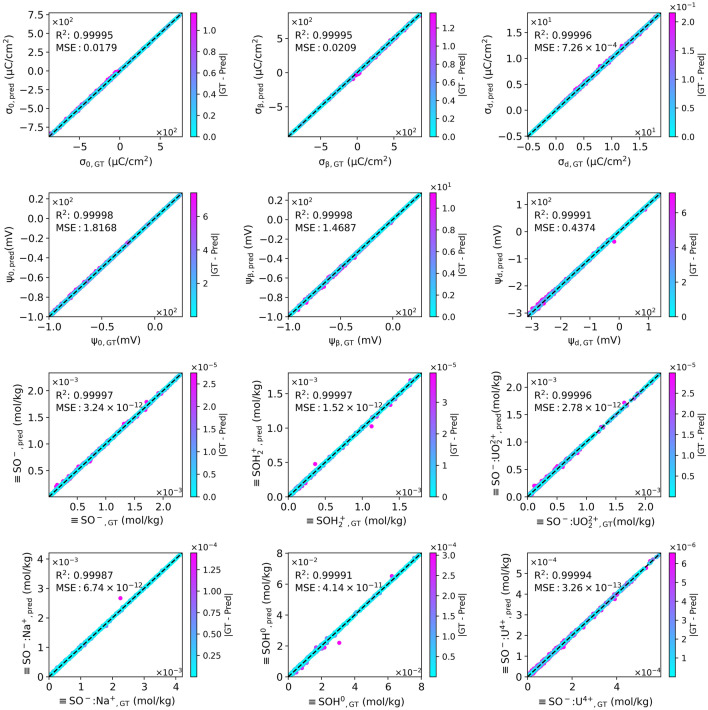
Performance of DNN-surrogate for uranium sorption on oxides. Parity plot for DNN-surrogate model prediction vs. numerically calculated ground truth using GWB. $R^2$ score represents the goodness of fit of surrogate predictions to corresponding ground truth values. MSE represents the mean squared error between prediction and target. The absolute error (|GT - Pred|) between DNN-surrogate prediction and the corresponding ground truth value is shown as the color bar.

We then trained both models on 1,271,528 data. The performance of RF-surrogate on predicting uranium sorption to oxide surfaces was evaluated by simultaneously testing the remaining 317883 conditions (input entries). For the Deep Neural Network surrogate model (DNN-surrogate), 158,941 data points from the remaining data were allocated for hyperparameter tuning and to mitigate overfitting. The model performance was then assessed on the remaining 158,942 conditions. A more detailed description of the model construction and dataset can be found in the “Methods” section.

Figure 2 is the parity plot of DNN-surrogate, which compares the charge densities, electrostatic potentials at different layers, and surface adsorption from numerical SCM solver to machine-learned results under diverse conditions. The overall $R^2$ score of RF-surrogate consistently exceeds 0.999 for all targets (see Supplementary Information, Fig. S1), whereas DNN-surrogate consistently achieves $R^2$ scores higher than 0.9999. Although RF-surrogate also received a remarkable value of $R^2$. The DNN surrogate improves upon the RF surrogate, with fewer outliers and more minor prediction errors, especially for surface sorption and charge density/electrostatic potential at the diffuse layer. The maximum absolute error of normalized data between the machine-learned prediction and the numerical SCM solver’s solution is approximately 0.015 for DNN-surrogate and around 0.04 for RF-surrogate. Utilizing DNN-surrogate has resulted in a 62.5% improvement in accuracy. We also calculated each target’s mean squared error (MSE) for each target variable while preserving the original units of the data, as displayed in Fig. 2. Compared to RF-surrogate, DNN-surrogate decreases the mean squared error (MSE) by 56.64–89.2% for each target.

We conducted two additional tests to assess the surrogate model’s performance further. We randomly generated two titration sets of input entries (see Table S3, Supporting Information) to compare the surrogate model’s and numerical solver’s predictions regarding pH-dependent charge densities and electrostatic potentials.

**Figure 3:**
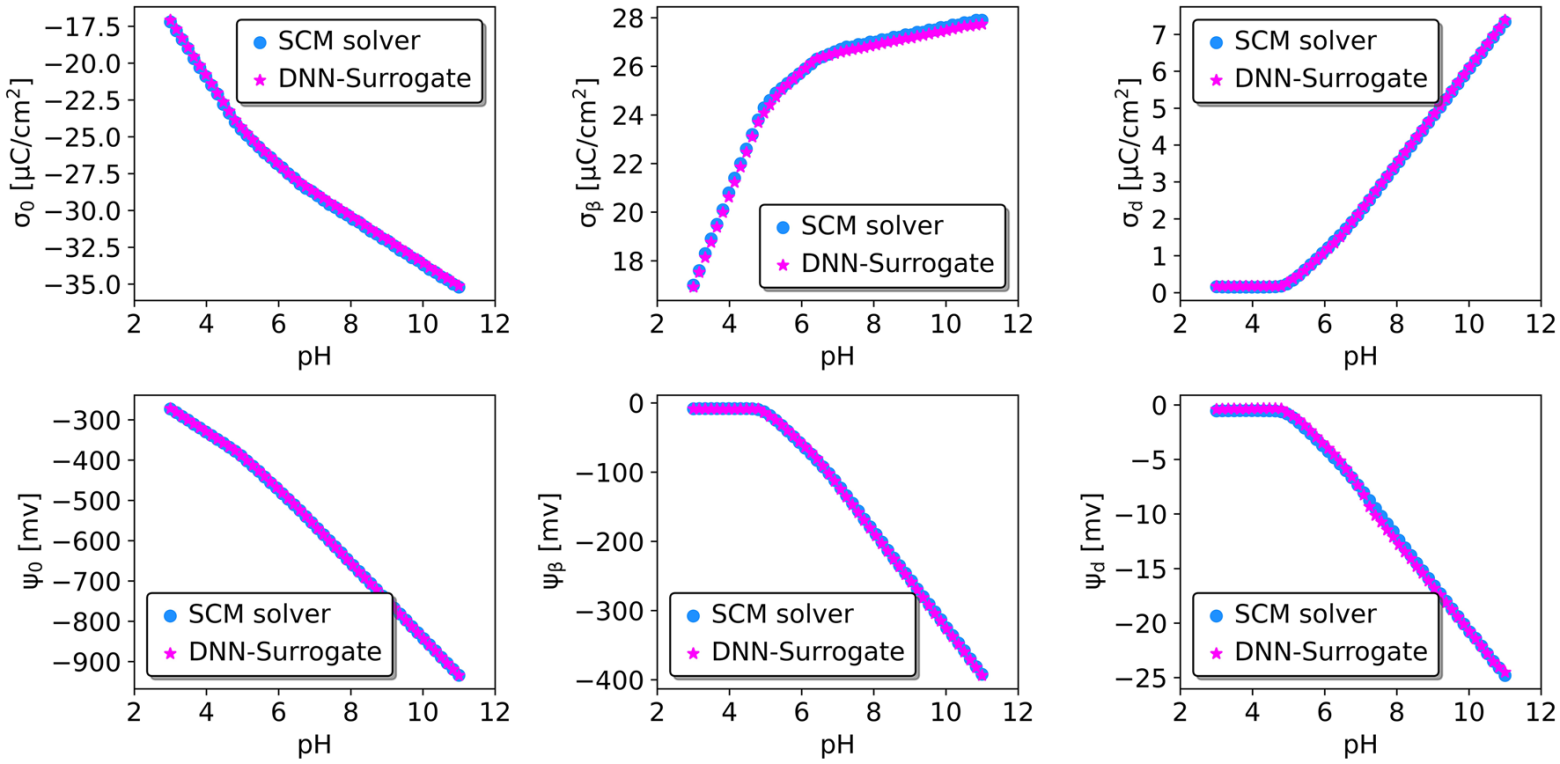
Comparison of charge densities and potentials at the surface, $\beta$, and diffuse layers predicted by DNN-surrogate’s predictions and those obtained by fully converged numerical SCMs.

DNN-surrogate can produce identical results as numerical SCM solvers for charge densities and electrostatic potentials regardless of locations (Fig. 3, first set). On the other hand, RF-surrogate generates similar results for $\beta$ layer charge density and diffuse layer potential (see Supplementary Information, Fig. S2), but fails to predict other targets accurately.

**Figure 4:**
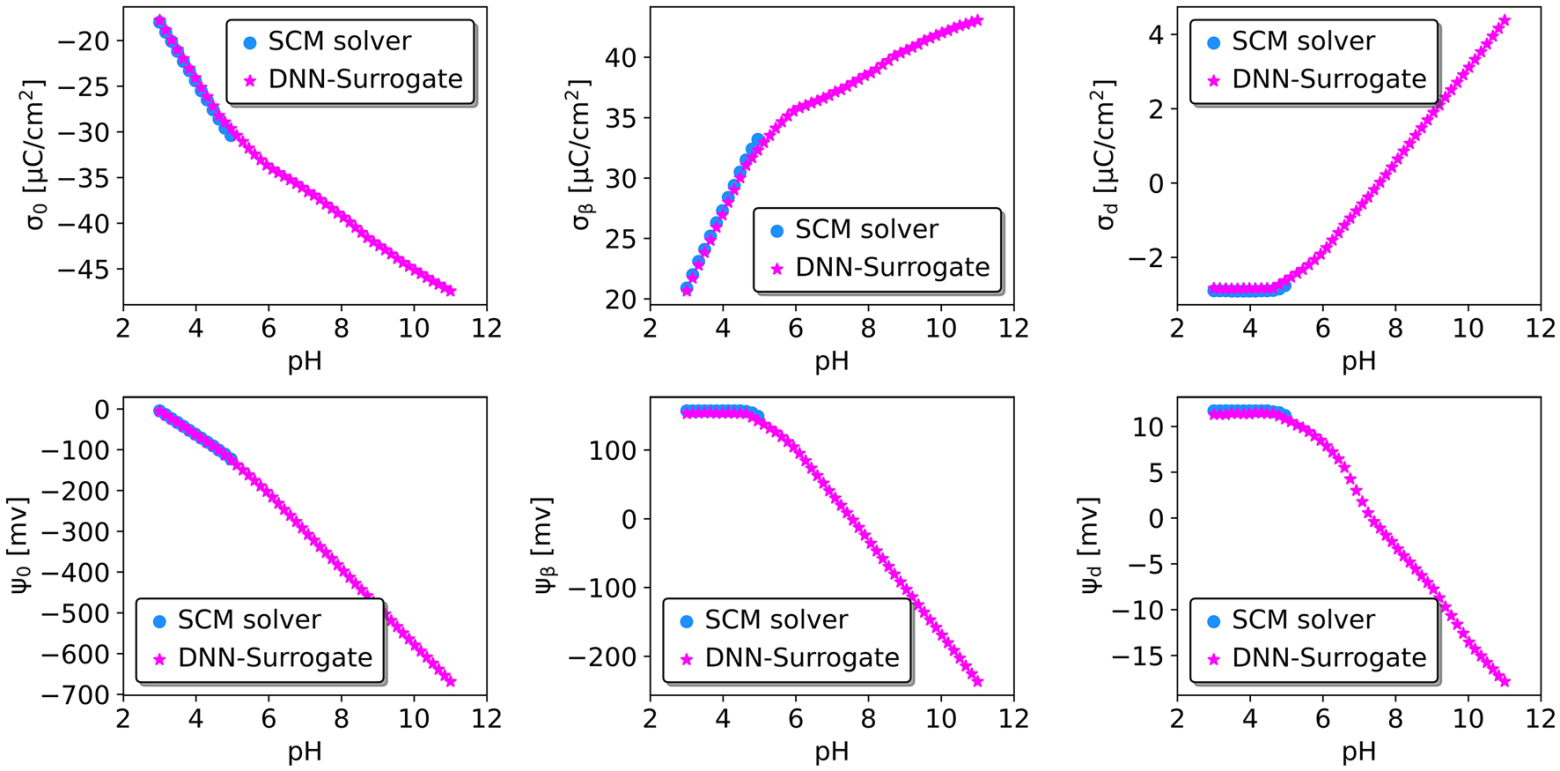
Comparison of charge densities and potentials at the surface, $\beta$, and diffuse layers predicted by DNN surrogate with those obtained by the numerical solver that encounters converge issues. Note that DNN-surrogate can provide predictions for all pH values in contrast to numerical SCM.

In the second test, we choose a set of parameters for which the numerical solver encounters convergence problems for pH above 5 (Fig. 4). In contrast, both surrogate models can produce the results for the whole pH range. Specifically, DNN-surrogate (but not RF-surrogate) consistently delivers reliable predictions for targets below and above the threshold of pH equal to 5. What is more, RF-surrogate cannot extrapolate when pH is beyond 5 (see Supplementary Information, Fig. S3).

To better understand how the DNN-surrogate model learned to map an input to output, we performed feature analysis by measuring how each input feature impacts each output value. Here, we implemented the integrated gradient algorithm^28^ to calculate the feature attribution by assigning an importance score to each input feature via approximating the integral of the gradients of the model’s output concerning the inputs.

For surface, $\beta$-layer, and diffuse layer charge densities, the top five crucial input features consistently include the uranium initial concentration in the solution, anions binding constant $\hbox {logK}_a$, pH, protonation equilibrium constant $\hbox {logK}_1$, and inner-layer capacitances $c_1$. By definition, the surface charge is the sum of positive and negative charges at the surface, the charge at $\beta$-plane is determined by accumulated charges in $\beta$-plane, and charge density at the diffuse layer is highly correlated to the charge density at the surface and $\beta$-plane, can be calculated via $\sigma _0 + \sigma _{\beta } + \sigma _d = 0$^29–33^. Therefore, it is reasonable for the DNN surrogate to discover that pH conditions, ion concentrations, and affinity constants that control the uptake and release of ions and protons are essential parameters for all charge density predictions.

The electrostatic potentials are coupled to the charge densities in the SCM. First, $c_1$ directly links the charge at the surface with the potential drop at a distance $\beta$ with the equation: $\sigma _0 = c_1(\psi _0 - \psi _{\beta })$^31^. Regarding feature importance, we anticipate similar patterns for $\psi _0, \psi _{\beta }$ with surface charge density $\sigma _0$. Analysis results reveal that $c_1$ is always the fifth important feature for predicting $\sigma _0, \psi _0, \psi _{\beta }$, and the top five most important features of potential near the surface (0 and $\beta$ layers) are in agreement with surface charge density, having the most relevant features being pH, [$\hbox {U}^{4+}$], affinity constants, and $c_1$. For diffuse layer potential, it follows the relation: $\sigma _d = c_2 (\psi _d - \psi _{\beta })$. Therefore, it is not surprising that the importance of $c_1$ decreased. In literature^29^, $c_2$ is often set to a constant value; hence, it is not considered in our dataset. Thus, an inconsistent feature importance between $\psi _d$ and $\psi _{\beta }$, $\sigma _d$ is expected.

The extent of accessible binding sites for surface sorption is directly governed by surface area. The analysis underscores the essential role of surface area as a critical feature for predicting surface reactions using DNN-surrogate. The analysis confirms the importance of affinity constants in accurately predicting surface reactions. For instance, equilibrium constants are key features in predicting proton/deprotonation processes and ion complexation reactions, and logK_UO22+_ and logK_U4+_ play crucial roles in predicting $\hbox {UO}_2^{2+}$ and $\hbox {U}^{4+}$ sorption to mineral surfaces, respectively (as expected based on assumed chemistry in the SCM construction, see “Methods” section).

**Figure 5:**
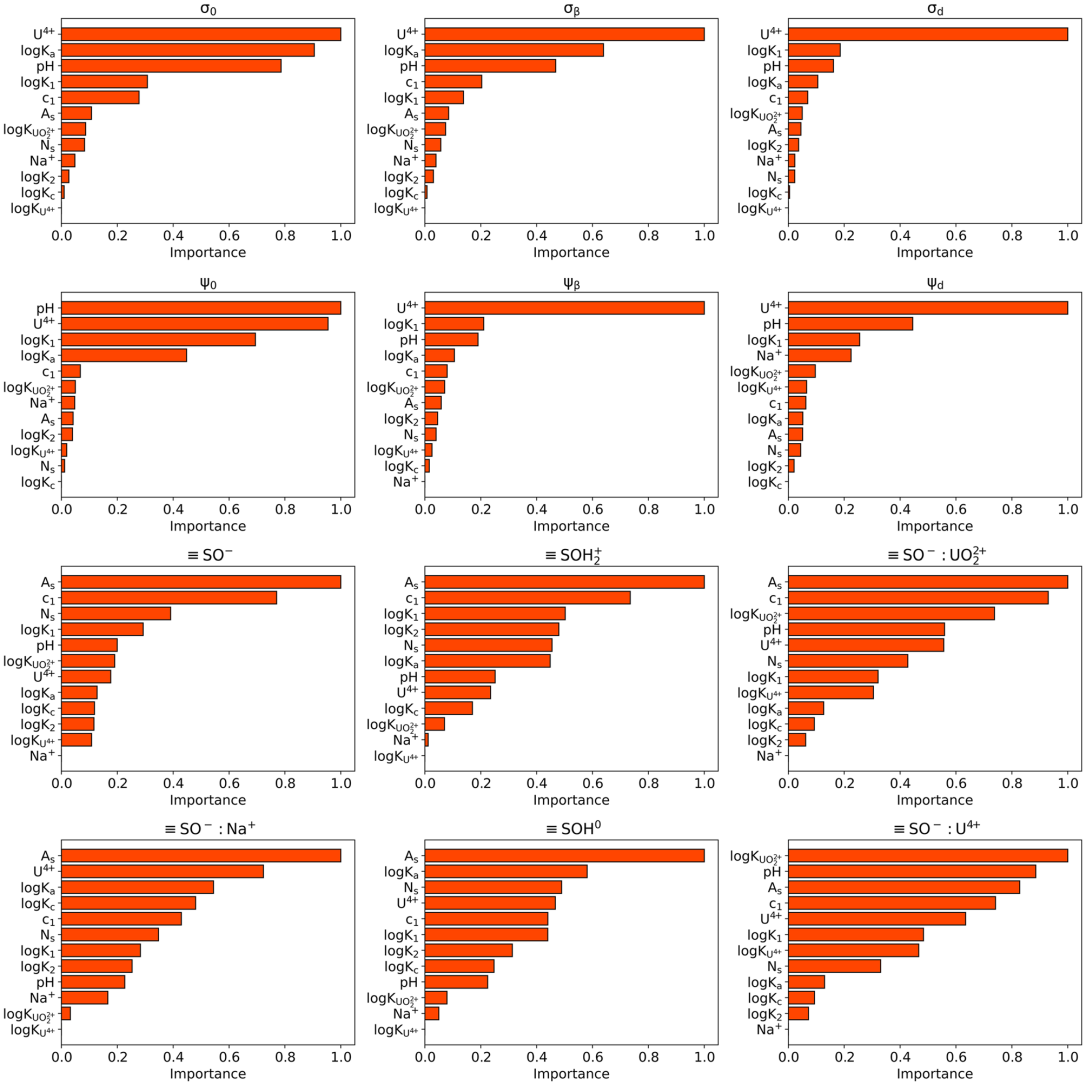
Analysis of input feature attribution in predicting target values for DNN-surrogate.

SCM equations are highly non-linear, and often, solutions show a convoluted interdependence between parameter values and predicted properties. Here, we identify several such correlations (refer to Fig. S6). The SCM construction imposes coupling between the electrostatic properties at various layers. Here, by analyzing training datasets, we noticed strong correlations between diffusion layer charge ($\sigma _d$) and the $\beta$ layer potential ($\psi _{\beta }$); between the surface charge ($\sigma _0$) and the $\beta$ layer charge ($\sigma _{\beta }$). One can expect similar correlations in the results of the feature importance analysis of these targets. As illustrated in Fig. 5, $\sigma _0$ and $\sigma _{\beta }$ exhibit nearly identical feature importance trends, differing only in the rank reversal of $\hbox {logK}_1$ and $\hbox {c}_1$. In the case of $\sigma _d$ and $\psi _{\beta }$, they share the same top eight features and trends, while the remaining input features, $\hbox {N}_s$, logK*c*, $\hbox {Na}^+$, and logK_U4+_, display distinct importance trends and are all less significant in accurately predicting the targets. However, the feature analysis of RF-surrogate (Fig. S6) reveals that only the initial four important features align for $\sigma _d$ and $\psi _{\beta }$, and the fourth and last three input features are consistent for $\sigma _0$ and $\sigma _{\beta }$. Merely 4 of 12 input features display analogous importance in strongly correlated pairs. In conclusion, the DNN-surrogate successfully recognized the underlying physicochemical relationships from training data, whereas the RF-surrogate only partially captured these relationships.

Table S1 summarizes the computational efficiency and utilized computing resources for three approaches in estimating uranium sorption under over 300,000 conditions. AI surrogates exhibit exceptional speed, making simultaneous predictions for all 300,000 conditions in seconds: 27 s for DNN-surrogate and 86 s for RF-surrogate. In contrast, traditional numerical SCM solvers need to solve for these conditions sequentially, taking over 5 h.

## Conclusion

The safety assessments of geological repositories for spent nuclear fuel require knowledge of the radionuclide transport and retention by surrounding rocks in case of a catastrophic event of leakage. If U(IV), a dominant form in reducing conditions, is transported upwards, it can be oxidized to a more mobile U(VI).

Here, we investigated two distinct AI surrogates for the SCM of uranium retention by oxides. We modeled various minerals varying in acid-base properties, site densities, surface area, and ion affinities for different ion concentrations (including uranium). We used the 2-pK TLM model of uranium sorption on the oxide/electrolyte interface, which was solved numerically by the GWB solver, to generate over a million entries for training AI models.

The DNN surrogate does not experience any convergence issues as compared to the numerical solver and RF surrogate, and it can make predictions at a fraction of the computational time required for the SCM solver. Moreover, DNN-surrogate outperformed RF-surrogate regarding the range of applicability and accuracy. The feature analysis showed that DNN-surrogate captured how uranium sorption depends on the system descriptors and model parameters—demonstrating that DNN learned the physicochemistry of SCM construct to some extent directly from the data.

Here, we showed how to construct a synthetic dataset and trained AI to mimic the 2-pK TLM model of uranium sorption. Our surrogates are the first step toward constructing an AI-based framework for modeling radionuclide subsurface migration that is essential in the safety assessment of geologic nuclear waste repositories. More extensive and complex chemistries will be examined in the future.

## Methods

### U(VI) SCM surrogate model development

The first step towards developing our U(VI) SCM surrogate is constructing the database of input-output pairs using an existing SCM solver. Here, we used the robust SCM solver provided by Geochemist’s Workbench (GWB) software^27^ because it is one of the most frequently used software in geochemical modeling. We also used the random forest algorithm (RF) and deep neural networks (DNN) for the surrogate model development, because RF is simple and efficient in classification and regression tasks^34^, and DNN is effective for making data-driven decisions^35^.

#### Data set generation

SCMs consist of chemical reactions characterizing the mineral surface charging process and ion complexation combined with geometric models of spatial charge distribution at the interface. To model the mineral surfaces, we used a generic oxide surface covered by reactive amphoteric surface sites ($\equiv$ SOH). These sites can act as an acid or a base by either releasing a proton or binding an additional proton. The acid-base chemistry of the surface group is described using the 2-pK protonation model^36–38^:

1 $$\begin{aligned} & \equiv \mathrm{SOH}_2^+ \quad \rightleftharpoons \quad \equiv \mathrm{SOH}^0 +\mathrm{H}^+ \qquad \text {and} \quad \mathrm{K}_1 = \frac{ \{\equiv \mathrm{SOH}^0 \} \{ \mathrm{H}^+ \}}{ \{ \equiv \mathrm{SOH}_2^+ \} } \end{aligned}$$

2 $$\begin{aligned} & \equiv \mathrm{SO}^- +\mathrm{H}^+ \quad \rightleftharpoons \quad \equiv \mathrm{SOH}^0 \qquad \text {and} \quad \mathrm{K}_2 = \frac{ \{ \equiv \mathrm{SOH}^0\} }{ \{ \equiv \mathrm{SO}^- \} \{ \mathrm{H}^+ \}} \end{aligned}$$

where $\equiv \mathrm{SOH}^0$, $\equiv \mathrm{SOH}_2^+$ and $\equiv \mathrm{SO}^-$ represent charge-neutral, positively and negatively charged sites, respectively.

The electrolyte and uranium ions accumulate near the surface as outer-sphere complexes in the $\beta$ layer (Fig. 1b). The ions accumulation is described as surface complexation. Here, we used notation adopted by Sverjensky^29^ and describe ions accumulation via the following desorption reactions:

3 $$\begin{aligned} & \equiv \mathrm{SO}^-\mathrm{C}^+ + \mathrm{H}^+ \quad \rightleftharpoons \quad \equiv \mathrm{SOH}^0 + \mathrm{C}^+ \qquad \text {and} \quad \mathrm{K}_\mathrm{C} = \frac{ \{ \equiv \mathrm{SOH}^0\} \{ \mathrm{C}^+ \} }{ \{ \equiv \mathrm{SO}^-{\mathrm{C}^+} \} \{ \mathrm{H}^+ \}} \end{aligned}$$

4 $$\begin{aligned} & \equiv \mathrm{SOH}_2^+ \mathrm{A}^- \quad \rightleftharpoons \quad \equiv \mathrm{SOH}^0 + \mathrm{A}^+ + \mathrm{H}^+ \qquad \text {and} \quad \mathrm{K}_\mathrm{A} = \frac{ \{ \equiv \mathrm{SOH}_2^+ \mathrm{A}^- \} }{ \{ \equiv \mathrm{SOH}^0 \} \{ \mathrm{H}^+ \} \{ \mathrm{A}^- \}} \end{aligned}$$

5 $$\begin{aligned} & \equiv \mathrm{SO}^-\mathrm{U}\mathrm{O}_2^{2+} + \mathrm{H}^+ \quad \rightleftharpoons \quad \equiv \mathrm{SOH}^0 + \mathrm{UO}_2^{2+} \qquad \text {and} \quad \mathrm{K}_{\mathrm{UO}_2^{2+}} = \frac{ \{ \equiv \mathrm{SOH}^0 \} \{ \mathrm{UO}_2^{2+} \} }{ \{ \equiv \mathrm{SO}^-\mathrm{UO}_2^{2+} \} \{ \mathrm{H}^+ \}} \end{aligned}$$

6 $$\begin{aligned} & \equiv \mathrm{SO}^-\mathrm{U}^{4+} + \mathrm{H}^+ \quad \rightleftharpoons \quad \equiv \mathrm{SOH}^0 + \mathrm{U}^{4+} \qquad \text {and} \quad \mathrm{K}_{\mathrm{U}^{4+}} = \frac{ \{ \equiv \mathrm{SOH}^0 \} \{ \mathrm{U}^{4+} \} }{ \{ \equiv \mathrm{SO}^-\mathrm{U}^{4+} \} \{ \mathrm{H}^+ \}} \end{aligned}$$

The electric field generated by a charged mineral surface affects ions ion concentrations near the surface. SCMs account for this effect by weighting bulk concentrations with electric-field energy terms as follows:^21,26,36–38^

7 $$\begin{aligned} \{\mathrm{X}^{\mathrm{z}_{\mathrm{x}}} \} = [\mathrm{X}^{\mathrm{z}_{\mathrm{x}}}] \exp \left( \frac{-\mathrm{z}_{\mathrm{X}} \mathrm{e} \psi _{\mathrm{j}} }{\mathrm{k}_{\mathrm{B}} \mathrm{T}} \right) \end{aligned}$$

where $\{\mathrm{X}\}, [\mathrm{X}]$ represents the interfacial and bulk concentrations of ion X, the electrostatic potential $\psi _j$ is equal to the surface potential for $\hbox {X}=\hbox {H}^+$ or $\beta$-layer potential for $\hbox {X}=\hbox {C}^+$, $\hbox {A}^-$, $\hbox {UO}_2^{2+}$, U^4+^ (see Fig. 1b), $z_X$ is the formal ion charge, $k_B$ is the Boltzmann constant and *T* is the temperature.

By solving the SCM model described above, one can determine concentrations of all types of surface complexes, charge densities ($\sigma _0, \sigma _\beta , \sigma _d$), and potentials ($\psi _0, \psi _\beta , \psi _d$) at the surface, $\beta$- and diffuse layers (see Fig. 1b). A detailed discussion of the SCM equations and numerical methodology can be found elsewhere^21–23,26^.

**Figure 6:**
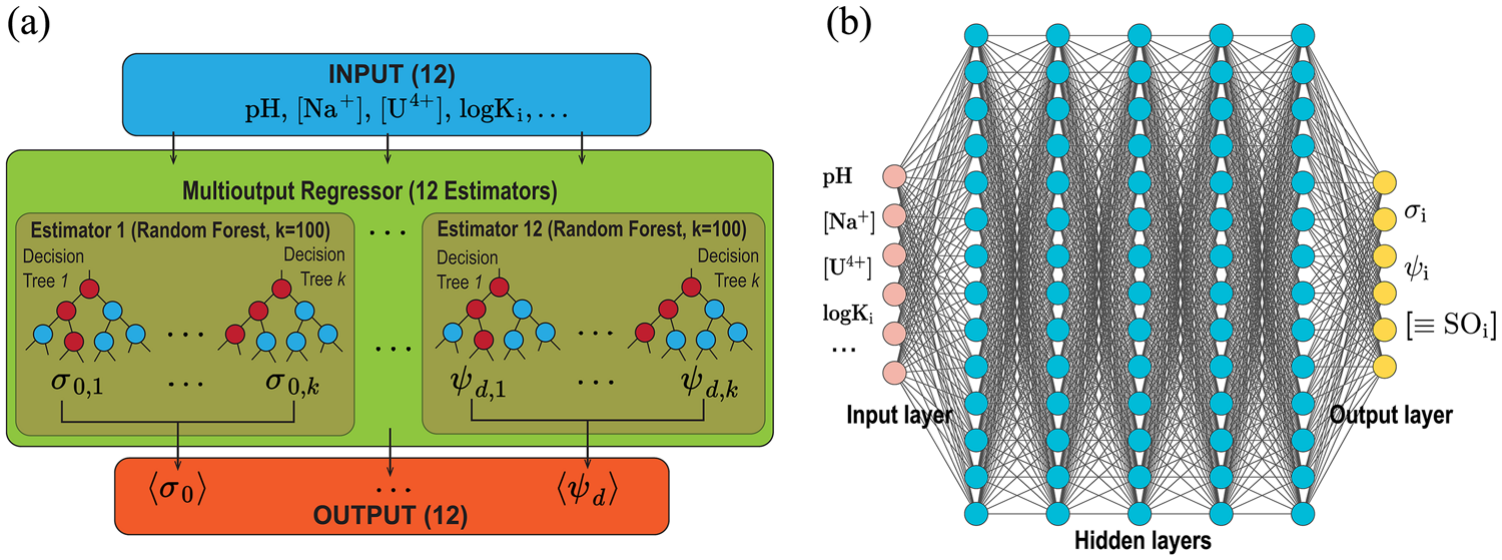
Illustration of AI Surrogates for Uranium Sorption on Mineral Surfaces: (**a**) RF-surrogate, a multioutput regressor as a SCM surrogate, comprising of 12 random forests (estimators), each trained to predict a single target value (output) based on a complete input vector. (**b**) DNN-surrogate, a deep neural network used as a surrogate model for SCM, consists of 5 hidden layers with 512 neurons per layer. Both surrogate models use the same input/output format. The input consists of a description of the oxide surface (surface area, site density), the SCM parameter values, such as affinity constants ($\hbox {logK}_i$) capacitances, and a description of solution composition (12 values). The output consists of charge density and electrostatic potential in each layer and concentrations of surface complexes (12 values).

The uptake of U(VI) by mineral surface depends on the surface charge of oxide at a given pH condition^20^. The non-specific adsorption is driven mainly by electrostatic interactions; in the case of uranium ions, they are attracted to positively charged cations by negatively charged surfaces. Consequently, uranium sorption occurs if pH exceeds the Point of Zero Charge (PZC) and generally increases with increasing pH. However, in the case of specific sorption, or sorption of $\hbox {UO}_2^{2+}$ complexed with $\hbox {Cl}^-$, U(VI) accumulation can occur even at pH below PZC (see Supplementary Information, Fig. S1). To generate an extensive dataset that covers most situations as much as possible for the ML training, we developed a Python script that modifies the uranium thermodynamic database by varying the environmental conditions, such as pH and salt concentrations. We also modified model parameters describing acid-base properties of the surface, surface site density, surface area, capacitance, logK values, and electrolyte/uranium affinity to the mineral surface. The ranges of explored inputs parameter space and environmental conditions are listed in Supplementary Information, Table S2. Consequently, we generated over 1.5 million data by fully exploring the range of uranium sorption conditions. To prepare the generated dataset for the random forest algorithm, we extracted the modified inputs (12) in the SCM solver and the uranium thermodynamic database and their subsequent outputs (12) (Fig. 6).

We split the database of extracted input parameters and output values described above into training and test sets with a ratio of 80:20 % for RF-surrogate and training, validation, and test sets of 80:10:10 % for DNN-surrogate. The min-max normalization strategy is applied to the data to improve training efficiency. We trained the model on the training dataset and evaluated its performance on the test set, which was never used during the training process.

#### Surrogate model construction and training strategy

We used Scikit-learn^39^ library for RF-surrogate’s model development, training, and testing. As previously^26^, we used the MultiOutputRegressor module from Scikit-learn to estimate multiple output values. A Random Forest Regressor was used as the base estimator for predicting each target, and a multioutput regressor consisting of 12 random forest regressors was implemented to predict 12 target values. Each random forest consists of 100 decision trees (Fig. 6). The RF-surrogate was trained on a single CPU for  1.5 h.

DNN-surrogate consists of 7 layers of interconnected nodes, including input, output, and 5 hidden layers. The number of neurons in each hidden layer is 512/512/512/512/512 respectively. Input and output dimensions are both 12 and stored in the same format as the one used in RF-surrogate (Fig. 6). During the training, we adopted Adam optimizer^40^ with default parameters for minimizing the loss function. A batch size of 128 was chosen; the initial value of the learning rate was set to 0.001 and was dynamically adjusted based on validation performance. Additionally, an early stopping strategy was implemented to reduce overfitting. The above selections of the number of hidden layers, optimization algorithms, and batch size are determined by RayTune framework^41^, which is a library for tuning the hyperparameters of deep learning models. The DNN-surrogate model was implemented in PyTorch^42^ and trained on a single NVIDIA A100 GPU for 2.3 h.

For both surrogate models, mean square error (MSE) was used to minimize the loss between the ground truth and the surrogate prediction during the training procedure.

The source code for our surrogate model is provided (see the “Data availability” section).

### Supplementary Information

Supplementary Information.

## Data Availability

The data used in this work is available for download from https://zenodo.org/records/10815543. Jupyter Notebook used to train the RF- and DNN-SCM models is available at: https://github.com/nodameCL/Uranium-Sorption-Surrogate.
