# Supplementary material for: Machine learning surrogates for surface complexation model of uranium sorption to oxides
Source: Sci Rep. 2024 Mar 19;14:6603. doi: 10.1038/s41598-024-57026-w (PMC10951217; doi:10.1038/s41598-024-57026-w)
Supplement: Supplementary file 1 — Supplementary Information. [file 41598_2024_57026_MOESM1_ESM.pdf]

Supplementary Information for: Machine Learning Surrogates for Surface Complexation Model of Uranium Sorption to Oxides

Chunhui Li[1,*], Elijah O. Adeniyi[2,*], and Piotr Zarzycki[1]

[1]Energy Geosciences Division, Lawrence Berkeley National Laboratory, Berkeley, California, USA
[2]Department of Earth Sciences, Montana State University, Bozeman, Montana, USA
[*]Correspondence: chunhuili@lbl.gov (CL) or elijahadeniyi@montana.edu (EA)

Contents

- Table S1. A summary of computational efficiency and utilized computing resource for numerical SCM solver, RF-surrogate, and DNN-surrogate.
- Table S2. The range of values of environmental conditions and model parameters are explored in this work.
- Table S3. Two additional datasets used to test model performance.
- Figure S1. Performance of RF-surrogate for uranium sorption on oxides.
- Figure S2. Comparison of charge densities and potentials at the surface, β layer, and diffuse layer between RF-surrogate's predictions and the numerical solver's solutions without convergence issues
- Figure S3. Comparison of charge densities and potentials at the surface, β layer, and diffuse layer between RF-surrogate's predictions and the numerical solver's solutions with convergence issues
- Figure S4. Analysis of input feature attribution in predicting target values for RF-surrogate.
- Figure S5. The effects pH on (a) charge density, (b) electrostatic potential, (c) Surface protonation and (d) ion adsorption.
- Figure S6. Correlation analysis on outputs of training data. (a) A dendrogram of target values. It divided features into different groups based on their relevance. The lower the height, the stronger the correlation. (b) Pair correlation between different target values.

Table S1. A summary of computational efficiency and utilized computing resource for numerical SCM solver, RF-surrogate, and DNN-surrogate.

	Computational Time (317883 data)	Computational resources
Traditional SCM solver	$\tilde{5}$ hours	1 CPU
RF-surrogate	86.71 seconds	1 CPU
DNN-surrogate	27.94 seconds	1 GPU

Table S2. The range of values of environmental conditions and model parameters are explored in this work.

	Parameter/Variable											
	c_1 (F/m^2)	N_s (sites/nm^2)	A (m^2/g)	U^{4+} (mol/kg)	Na^+ (mol/kg)	pH	logK_1	logK_2	logK_C	logK_A	log$K_{U(VI)}$	log$K_{U(IV)}$
min	0.6	10	10	1E-5	1E-5	2	-10	2.1	1.2	-13	-13	-13
max	1.4	20	300	0.1	1.6	12	0.9	15	15	-1.1	-1.4	-1.2

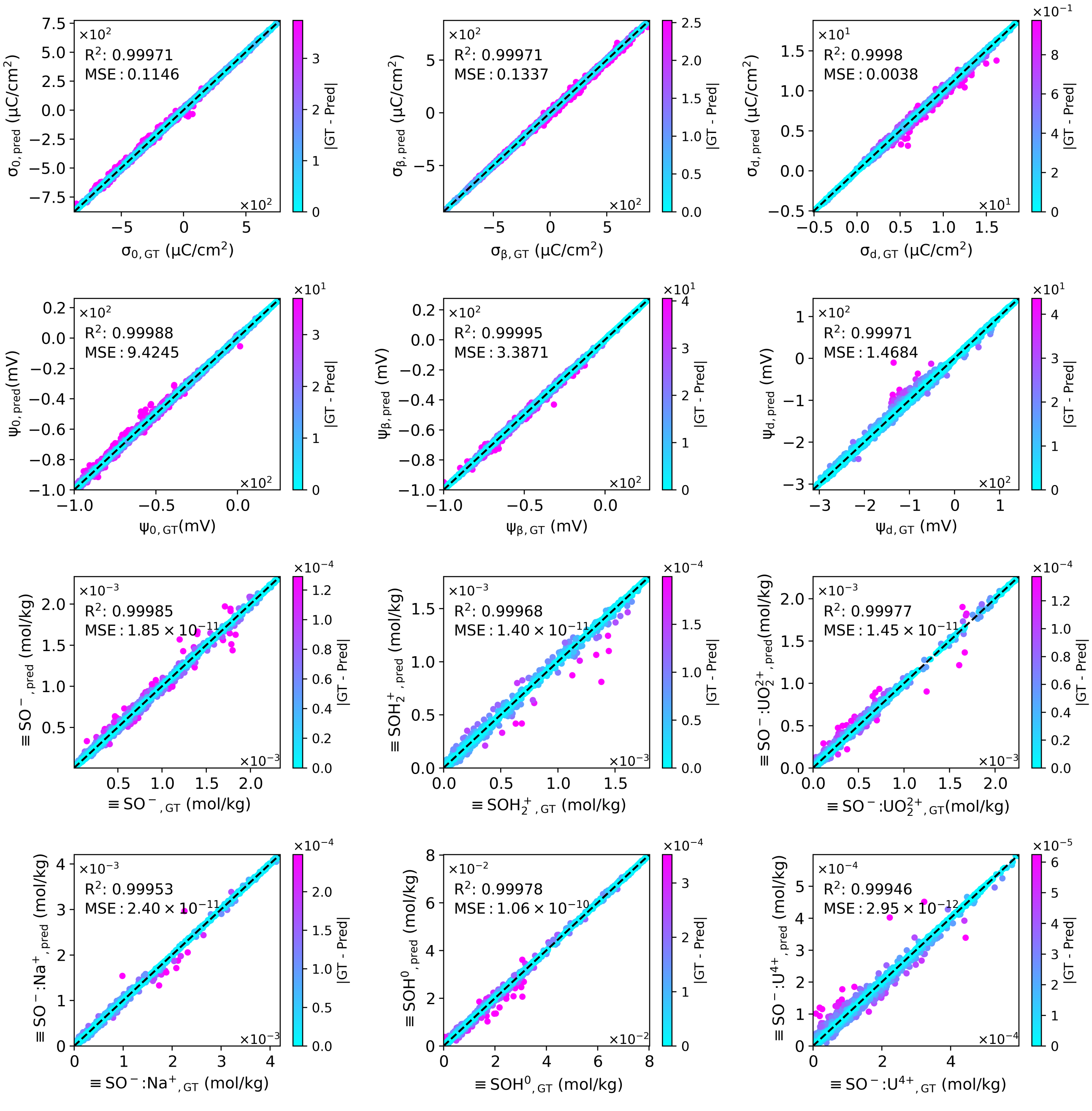

Figure S1. **Performance of RF-surrogate for Uranium Sorption on Oxides.** Parity plot for RF-surrogate model prediction vs. GWB calculated ground truth.R^2 score represents the goodness of fit of surrogate predictions to corresponding ground truth values. MSE represents the mean squared error between prediction and target. The absolute error between DNN-surrogate prediction and corresponding ground truth value is shown as the color bar.

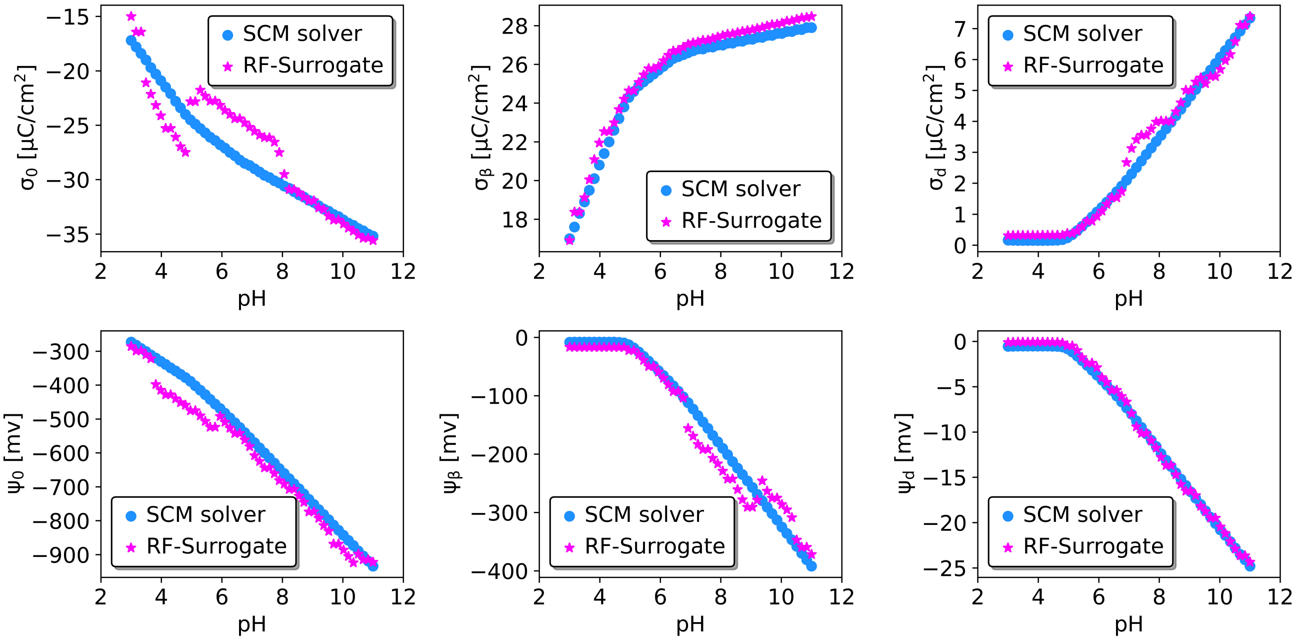

Figure S2. Comparison of charge densities and potentials at the surface, β layer, and diffuse layer between RF-surrogate's predictions and the numerical solver's solutions without convergence issues

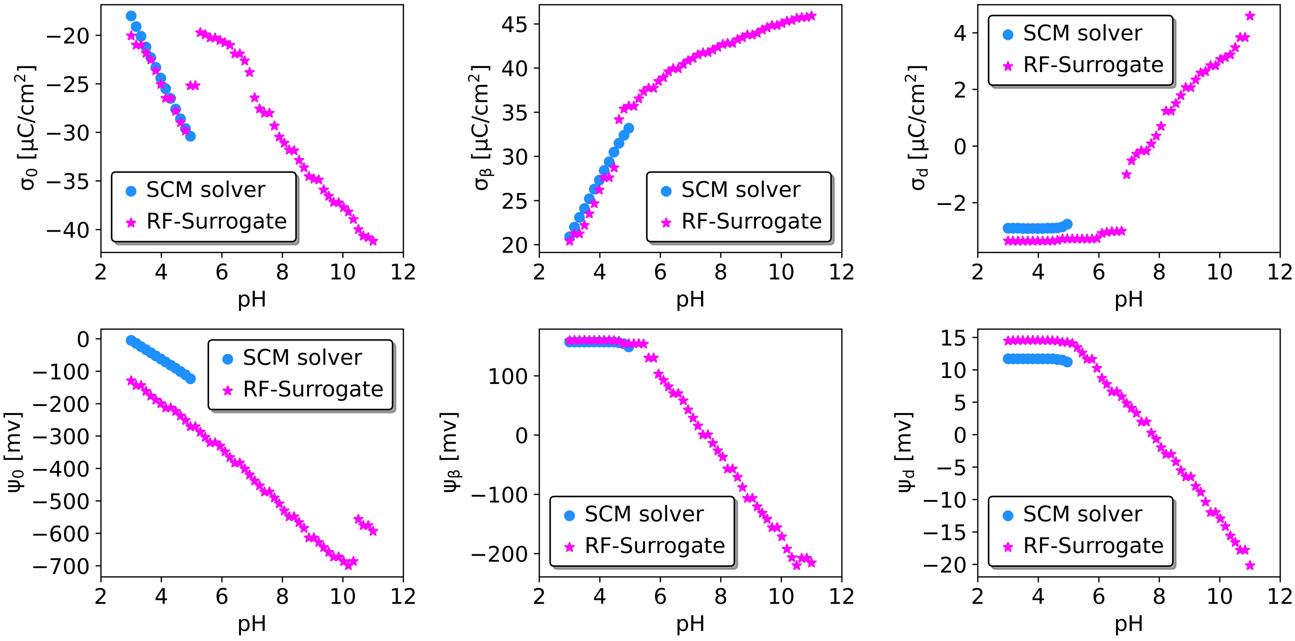

Figure S3. Comparison of charge densities and potentials at the surface, β layer, and diffuse layer between RF-surrogate's predictions and the numerical solver's solutions with convergence issues

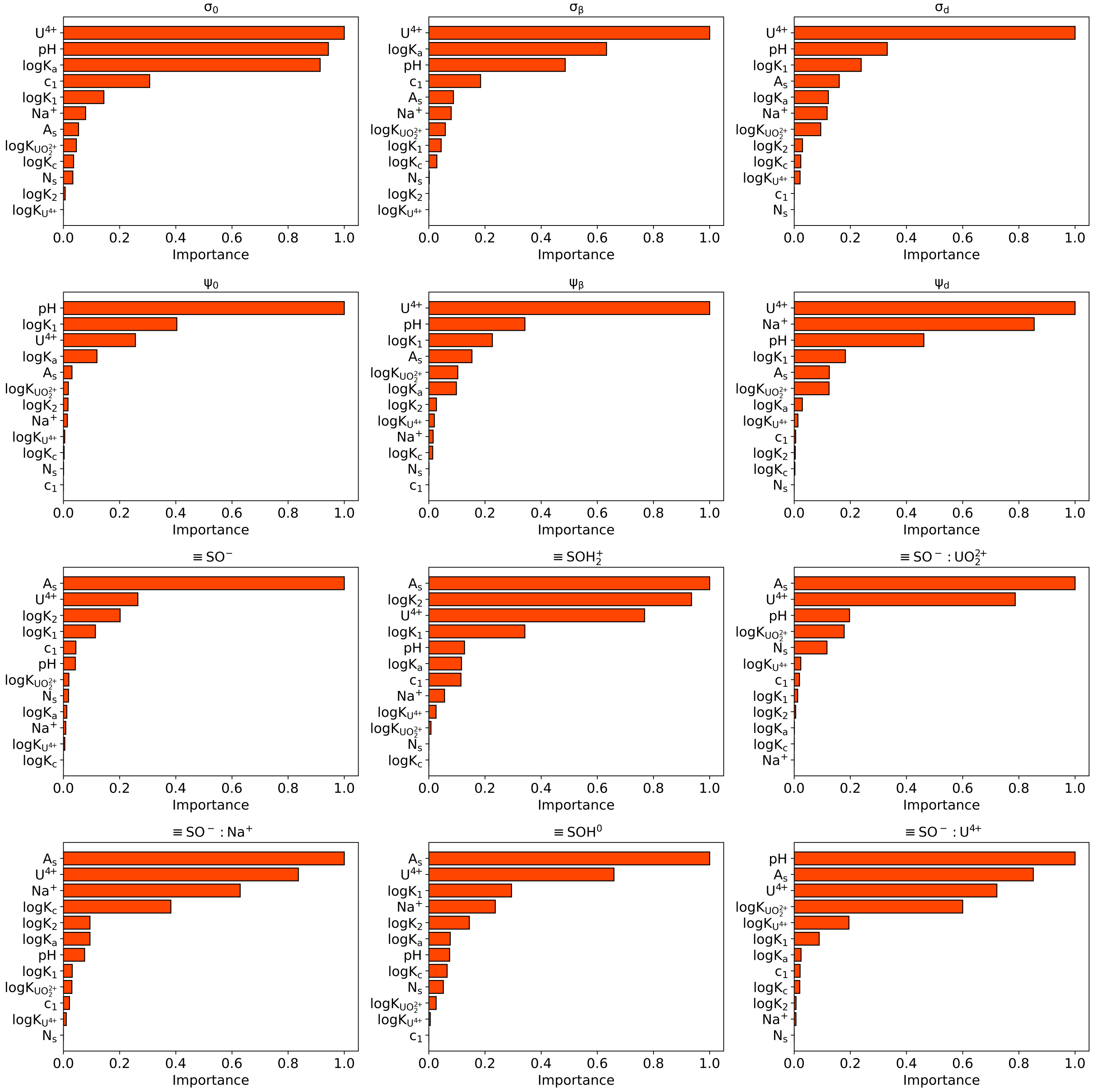

Figure S4. Analysis of input feature attribution in predicting target values for RF-surrogate.

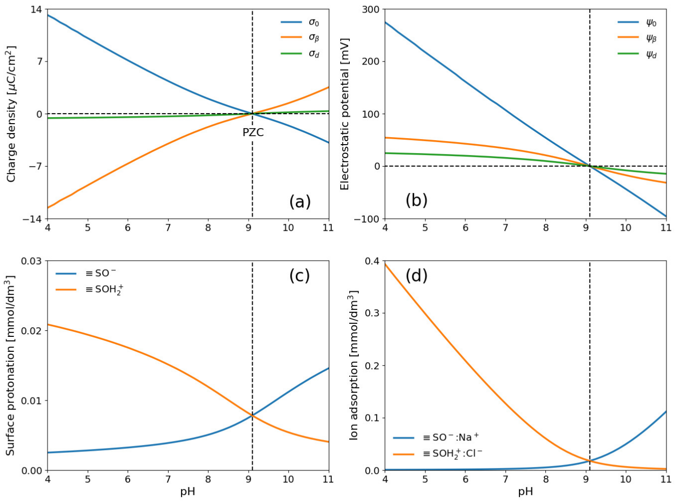

Figure S5. The effects pH on (a) charge density, (b) electrostatic potential, (c) Surface protonation and (d) ion adsorption.

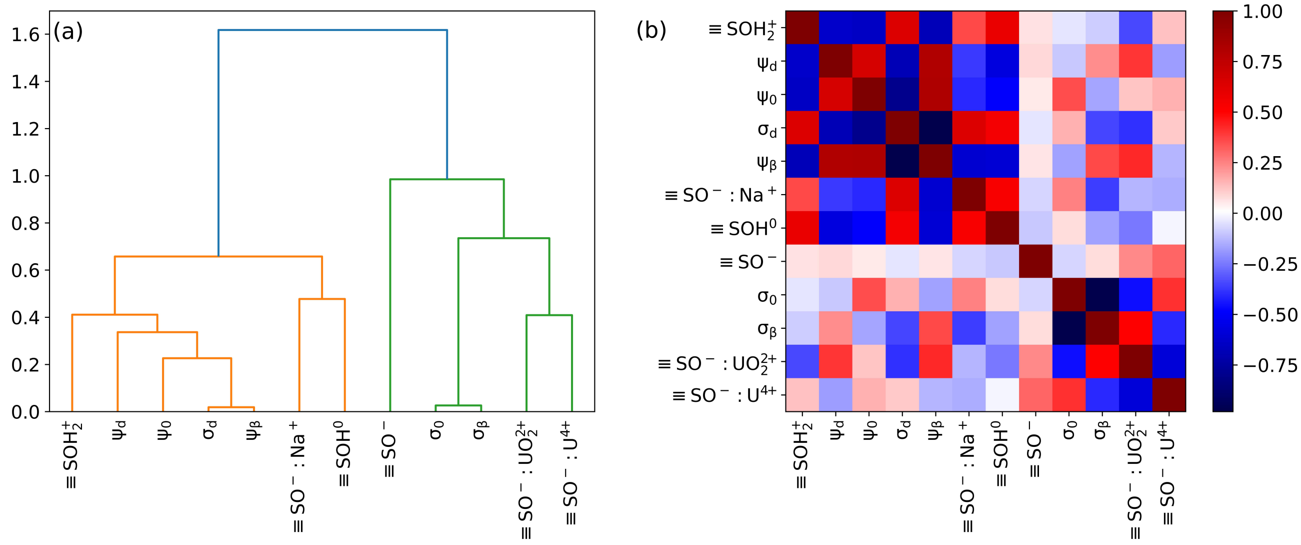

Figure S6. Correlation analysis on outputs of training data. (a) A dendrogram of target values. It divided features into different groups based on their relevance. The lower the height, the stronger the correlation. (b) Pair correlation between different target values.

Table S3. Two additional datasets used to test model performance. Set 1 is used in Figure 3, and set 2 is used in Figure 4.

	Parameter/Variable											
	c_1 (F/m^2)	N_s (sites/nm^2)	A (m^2/g)	U^{4+} (mol/kg)	Na^+ (mol/kg)	pH	$\log K_1$	$\log K_2$	$\log K_C$	$\log K_A$	$\log K_{U(VI)}$	$\log K_{U(IV)}$
set1	0.65	10	10	0.05	1.59	3-11	-7.08	14.59	14.19	-4.23	-10.58	-1.94
set2	1.12	15.96	84.47	0.087	1.09	3-11	-0.14	11.79	12.92	-3.73	-9.01	-12.23
